# P02-018 - PSTPIP1 gene mutations in periodic fever patients

**DOI:** 10.1186/1546-0096-11-S1-A125

**Published:** 2013-11-08

**Authors:** K Takabe, Y Adachi, H Saito, T Yamashita, Y Wakai, K Saito, Y Shinohara

**Affiliations:** 1 Internal Medicine, Tsuchiura Kyodo Hospital, Tsuchiura, Japan

## Introduction

Familial Mediterranean Fever (FMF) is considered a rare disease in Japan.

Our institution began screening for MEFV gene mutations in patients with periodic fever in 2005. Among the 18 patients screened, we have identified 11 (56.5%) FMF patients with heterozygous M694I/E148Q mutations. Among the other 7 patients, no pathogenic mutations were detected by the direct sequencing of all exons and the promoter region of the MEFV gene. PSTPIP1, the protein responsible for PAPA syndrome (pyogenic arthritis, pyoderma gangrenosum, and acne), has recently been found to bind with pyrin and to allow pyrin to interact with ASC. In this study we investigated whether PSTPIP1 mutations could be found in the 7 periodic fever patients without pathogenic MEFV mutations.

## Objectives

The patients were 3 males and 4 females with a mean age of 40.1 years (range: 5~74 years). The mean age at onset was 37.6 years (range: 2~72 years). The pattern of fever was consistent with the Tel-Hashomer criteria. The symptoms other than fever were headache (3 patients), pharyngeal pain (2), arthralgia (2), and skin rash (2). None of the patients experienced abdominal or chest pain during their fevers.

## Methods

We extracted DNA from peripheral leukocytes and performed sequence analyses of all exons and their flanking sequences of the PSTPIP1 gene. We also sequenced the MVK gene (exons 2 to 11), NFLP3 gene (exon 3), and TNFRSF1 gene (exons 2 to 4 and 6 to 7).

## Results

MEFV mutations identified in these patients were as follows; three heterozygous L110P/E148Q mutations, one heterozygous E148Q/R202Q and one P115R mutation. No mutations were detected in the MVK, NLRP3, or TNFRSF1 gene. One novel missense mutation (c.5 C>T, p.Met2Thr) was identified in exon 2. Single nucleotide variants in intronic regions were frequently found. A 14 bps deletion in intron 14 was detected in 3 patients and a 3 bps deletion was detected in the 3’ downstream region in 4 patients. The longer tandem repeats of (CCTG)8 in the promoter region were detected in 3 patients. None of the 43 healthy controls were positive for the p.Met2Thr mutation. A 14 bps deletion was detected in 3 of 42 healthy controls and a 3 bps deletion was detected in 20 of 42 controls. Six control cases were positive for the L110P/E148Q mutation, but none of them possessed a 14 bps deletion. In contrast, the three periodic fever patients with L110P/E148Q mutations also possessed 14 bps deletions. The frequency of 14 bps deletions in the L110P/E148Q mutation-positive cases with periodic fever was significantly higher than that in the L110P/E148Q mutation-positive cases without fever (healthy controls, COPD patients, and pulmonary fibrosis patients) (3/3 vs. 0/20, p=0.0006, Fishers’ exact test). RT-PCR analyses of exons 14 and 15 were performed using RNA extracted from two periodic fever patients with 14 bps deletions, but no splicing variants of exon 15 were identified.

## Conclusion

The p.Met2Thr variant was absent in the healthy controls, but its pathogenic role in the fever patients is unknown. The significance of the co-existence of L110P/E148Q mutation and 14 bps deletions also remains to be determined. Further investigations to elucidate how these sequence variants impact the pyrin/ASC interaction are awaited.

## Disclosure of interest

None declared.

